# Ancient pathogen DNA in human teeth and petrous bones

**DOI:** 10.1002/ece3.3924

**Published:** 2018-02-26

**Authors:** Ashot Margaryan, Henrik B. Hansen, Simon Rasmussen, Martin Sikora, Vyacheslav Moiseyev, Alexandr Khoklov, Andrey Epimakhov, Levon Yepiskoposyan, Aivar Kriiska, Liivi Varul, Lehti Saag, Niels Lynnerup, Eske Willerslev, Morten E. Allentoft

**Affiliations:** ^1^ Centre for GeoGenetics Natural History Museum of Denmark University of Copenhagen Copenhagen Denmark; ^2^ Institute of Molecular Biology National Academy of Sciences Yerevan Armenia; ^3^ Department of Bio and Health Informatics Technical University of Denmark Kongens Lyngby Denmark; ^4^ Peter the Great Museum of Anthropology and Ethnography (Kunstkamera) RAS St Petersburg Russia; ^5^ Samara State University of Social Sciences and Education Samara Russia; ^6^ Institute of History and Archaeology RAS (South Ural Department) South Ural State University Chelyabinsk Russia; ^7^ Russian‐Armenian University Yerevan Armenia; ^8^ School of Humanities Tallinn University Tallinn Estonia; ^9^ Department of Evolutionary Biology Institute of Molecular and Cell Biology University of Tartu Tartu Estonia; ^10^ Estonian Biocentre Institute of Genomics University of Tartu Tartu Estonia; ^11^ Department of Forensic Medicine Section of Forensic Pathology University of Copenhagen Copenhagen East Denmark; ^12^ Department of Zoology University of Cambridge Cambridge UK; ^13^ Wellcome Trust Sanger Institute Hinxton Cambridgeshire CB10 1SA UK

**Keywords:** ancient DNA, ancient pathogens, metagenomics, petrous bone, plague, *Yersinia pestis*

## Abstract

Recent ancient DNA (aDNA) studies of human pathogens have provided invaluable insights into their evolutionary history and prevalence in space and time. Most of these studies were based on DNA extracted from teeth or postcranial bones. In contrast, no pathogen DNA has been reported from the petrous bone which has become the most desired skeletal element in ancient DNA research due to its high endogenous DNA content. To compare the potential for pathogenic aDNA retrieval from teeth and petrous bones, we sampled these elements from five ancient skeletons, previously shown to be carrying *Yersinia pestis*. Based on shotgun sequencing data, four of these five plague victims showed clearly detectable levels of *Y. pestis* DNA in the teeth, whereas all the petrous bones failed to produce *Y. pestis* DNA above baseline levels. A broader comparative metagenomic analysis of teeth and petrous bones from 10 historical skeletons corroborated these results, showing a much higher microbial diversity in teeth than petrous bones, including pathogenic and oral microbial taxa. Our results imply that although petrous bones are highly valuable for ancient genomic analyses as an excellent source of endogenous DNA, the metagenomic potential of these dense skeletal elements is highly limited. This trade‐off must be considered when designing the sampling strategy for an aDNA project.

## 1 INTRODUCTION

Recently, a number of ancient DNA (aDNA) studies have provided significant insight into the prevalence and evolution of ancient human pathogens (Andam, Worby, Chang, & Campana, 2016; Bos et al., 2014; Bouwman et al., 2012; Devault et al., 2014; Harbeck et al., 2013; Maixner et al., 2016; Schuenemann et al., 2013). For example, Rasmussen et al. (2015) demonstrated the presence of the plague bacterium (*Yersinia pestis*) in early Bronze Age humans, at least 3,000 years before any historical recordings of this disease; Bos et al. (2014) identified seals as a likely source of New World human tuberculosis, and Schuenemann et al. (2013) described a high degree of genome conservation across 1,000 years of evolution in the leprosy‐causing *Mycobacterium leprae*. Considering the vast potential for such ancient disease studies, procedures that can optimize pathogen DNA recovery from skeletons are of great value.

DNA samples with a high endogenous DNA content (i.e., DNA from the target species) are crucial for ancient genomic studies because they can reduce the amount of required sequencing by orders of magnitude. For many ancient samples, the endogenous DNA content is very low (<1%), why recent aDNA efforts have aimed at maximizing the endogenous DNA content through improved sampling, DNA extraction, and library preparation protocols (Allentoft et al., 2015; Damgaard et al., 2015; Gamba et al., 2014; Gansauge & Meyer, 2014; Hansen et al., 2017; Korlević et al., 2015). The “discovery” of the petrous bone as an excellent aDNA resource represents a major improvement. Being the hardest bone in the mammalian body (Frisch, Sørensen, Overgaard, Lind, & Bretlau, 1998), the petrous part of the temporal bone contains high levels of endogenous DNA (Gamba et al., 2014), peaking in the otic capsule (Pinhasi et al., 2015) which is the highly compact bone surrounding the inner ear. Despite its obvious advantages in preserving endogenous DNA, ancient pathogen DNA has not, to our knowledge, been reported from petrous bone. This could simply be a coincidence or due to the fact that the compact bone structure of the otic capsule has a lower level of blood supply than other parts of the skeleton (Anson, Winch, Warpeha, & Donaldson, 1966), reflecting a much lower tissue turnover rate (Jørkov, Heinemeier, & Lynnerup, 2009). The petrous bone, however, is not the only skeletal part with high endogenous DNA content. When ancient teeth are well preserved, the cementum‐rich outer layer of the roots can yield levels of endogenous DNA that are often comparable to those of petrous bone (Hansen et al., 2017). Most ancient pathogens detected so far have been extracted from either postcranial bones or teeth, possibly because these have an abundant network of blood vessels/circulation and thus become directly exposed to blood‐borne pathogens.

Because ancient anthropological material available for aDNA studies is valuable and often highly limited, it is crucial to select skeletal elements and apply sampling techniques that fit the aim of the given project, maximizing the chance of a successful outcome. Therefore, we set out to compare the potential for pathogenic aDNA retrieval from the two most commonly used substrates in human aDNA studies, namely teeth and petrous bones. We sampled the petrous bones and teeth from five Bronze Age and Iron Age skeletons that have previously been shown to be carrying *Y. pestis* based on aDNA extracted from their teeth (Rasmussen et al., 2015). By resampling and “shotgun” sequencing these skeletons, we could assess the reproducibility of plague detection between teeth from the same individuals. It further allowed us to systematically test for differences in the presence of *Y. pestis* DNA in tooth cementum (outer hard layer of the root), tooth dentine (inner softer root layer), and the petrous bone. As negative control samples, we used data from teeth and petrous bones from 10 historical skeletons from a locality in Denmark allowing us to establish baseline levels and to test for more general differences in microbial DNA diversity in the different substrates.

## 2 METHODS

We resampled five Bronze Age and Iron Age human skeletons, previously revealed as being plague victims, owing to the presence of *Yersinia pestis* DNA extracted from their teeth (Rasmussen et al., 2015). In case of four individuals, we sampled both a petrous bone and a tooth, while only a tooth was available for the last individual. The tooth roots were separated into two fractions with a diamond blade: the outer cementum‐rich layer and the inner part, the dentine (facing the pulp cavity). For the four petrous bone samples, the otic capsule was targeted for DNA extraction (Pinhasi et al., 2015). A total of 14 sample fractions, that is, tooth cementum (*n* = 5), tooth dentine (*n* = 5), and petrous bone (*n* = 4), were DNA extracted in a dedicated aDNA laboratory at Centre for GeoGenetics, Natural History Museum, University of Copenhagen, according to strict aDNA standards (Willerslev & Cooper, 2005).

To maintain direct comparability with the results of the original *Y. pestis* work by Rasmussen et al. (2015), and with our negative control samples (see below), a brief “predigestion” step was implemented as part of the DNA extraction (Damgaard et al., 2015), despite the fact that such step may remove or dilute pathogen DNA on the sample surfaces while enriching for the endogenous human DNA. DNA extraction was performed with a silica‐in‐solution method, optimized at recovering very short DNA fragments (Allentoft et al., 2015). Next, 20 μl of DNA extract was prepared as blunt‐end double‐stranded libraries using Illumina‐specific adapters and NEBNext DNA Sample Prep Master Mix Set 2 (E6070) kit as described in Allentoft et al. (2015). Index‐amplified DNA libraries were purified and quantified on an Agilent Bioanalyzer 2100. The library pools were sequenced (80 bp, single read) on Illumina HiSeq 2500 platforms at the Danish National High‐throughput DNA Sequencing Centre. We also incorporated previously sequenced data (Hansen et al., 2017), representing teeth roots (cementum layer) and petrous bones from 10 historical age individuals from Holmens Kirke Churchyard (Denmark) as negative controls: samples H1‐H10 as in Hansen et al (2017). These samples have not previously been tested for plague but given their age and place of burial, there are no indications that these could be potential plague victims.

Two complementary methods were used for *Y. pestis* DNA detection: (1) mapping of DNA sequencing reads to an *Y. pestis* reference genome and (2) using Kraken—a taxonomic sequence classifier (Wood & Salzberg, 2014)—to identify *Y. pestis* DNA sequences among the reads. While the first method relies on mapping DNA sequences against the reference genome of the target species, allowing certain number of mismatches, Kraken finds exact matches of *k*‐mers (*k* = 31 by default) when comparing the sequences against a preassembled taxonomically classified k‐mers database.

We used AdapterRemoval 1.5.2 (Lindgreen, 2012) to remove adapter sequences and stretches of Ns at both ends of raw sequencing reads keeping only sequences with a minimum length of 30 bp. The trimmed sequences were mapped against the *Y. pestis* CO92 genome including the associated plasmids pCD1, pMT1, pPCP1 using bwa 0.6.2 aligner (Li & Durbin, 2009) with seeding disabled, allowing for higher sensitivity (Schubert et al., 2012). We kept only aligned DNA sequences with mapping quality of at least 30 and removed duplicates using Picard (http://broadinstitute.github.io/picard/). Kraken was run using default parameters (https://ccb.jhu.edu/software/kraken/MANUAL.html) based on a custom Kraken library containing all genomes of bacteria, fungi, archaea, protozoa, viruses, and humans in the RefSeq database (release 78), as well as all mitochondrial, plasmid, and plastid sequences as of November 2016. To visualize the results of the metagenomic analysis, we used Krona (Ondov, Bergman, & Phillippy, 2011).

Lastly, to estimate the endogenous human DNA content in these samples and determine mitochondrial DNA haplogroups (for comparison with the results on the same skeletons in the previous publications), we also mapped the trimmed DNA reads against the human reference genome build 37.1 with the same parameters as described above. The consensus human mtDNA sequence for each sample was called using a perl script that considers only bases with a base quality score of 20 and sites with a sequencing depth of at least 5×, and at each position, a base was called only if it was observed in at least 70% of the reads covering that site (Malaspinas et al., 2014). We used haplogrep to assign mitochondrial haplogroups of the ancient plague‐positive individuals (Kloss‐Brandstätter et al., 2011).

## 3 RESULTS AND DISCUSSION

We shotgun sequenced 14 samples representing five ancient human individuals, previously shown as being carriers of *Yersinia pestis* and added data from 20 putative negative control samples, representing 10 historical age human individuals (Table S1). In total, we analyzed 596,874,418 sequences, ranging from 2,399,751 to 42,244,695 per sample, averaging 17,555,130 (Table S1). All the sequences analyzed in this study have been submitted to the European Nucleotide Archive (https://www.ebi.ac.uk/ena) under study accession number PRJEB24937.

### 3.1 The mapping approach

**Figure 1:**
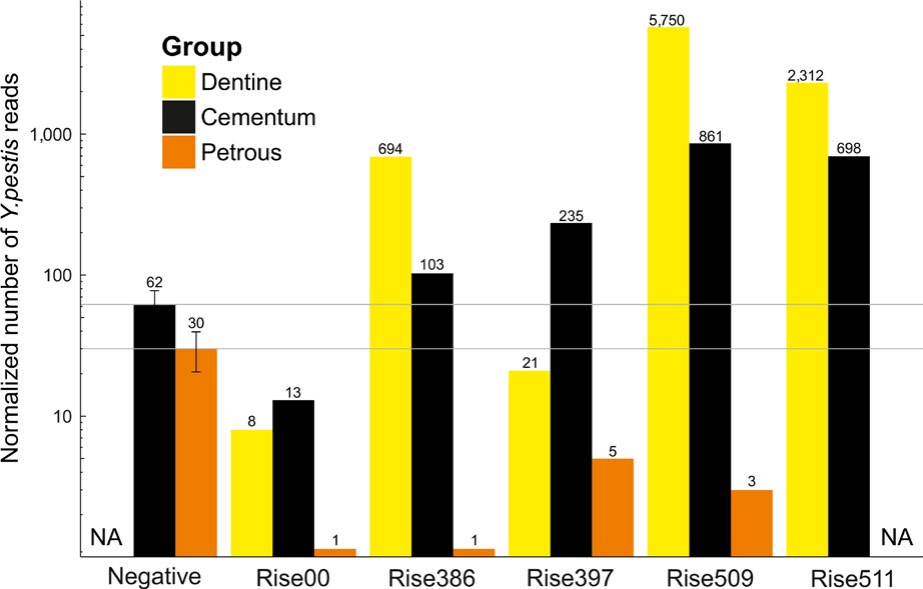
Normalized number of DNA sequences mapping to the *Y. pestis* reference genome. The DNA was extracted from teeth and petrous bones of five ancient skeletons known to be plague victims (Rise numbers) and 10 putative *Y. pestis* negative skeletons. Normalizations are based on the mapped fractions presented in Table 1 and assuming 10 million sequences pre‐mapping, which represent a typical average data output per library in a shotgun screening experiment (e.g., Allentoft et al., 2015). We note that these normalized numbers do not account for a potential slight reduction or increase in sequence clonality, as expected with reduced or increased sequencing effort. The reported values of the negative control samples are averages of 10 samples with 95% confidence intervals indicated (for teeth and petrous bones, respectively). The *Y* axis is in log scale; the gray horizontal lines represent average values of the negative samples

In the mapping analysis, the putative negative control samples displayed average sequence fractions of 6.2E^−6^ from teeth (*n* = 10) and 3E^−6^ from petrous bones (*n* = 10) mapping to the *Y. pestis* genome (Table 1). For the purpose here, we consider this to be a reasonable baseline approximation, reflecting background levels of nonspecific mapping of reads to the *Y. pestis* genome from other related taxa present in the environment. Despite sample‐to‐sample variance, Rise386, Rise397, Rise509, and Rise511 display *Y. pestis* DNA levels in the teeth that are clearly above the average background level (Table 1, Figure 1). Among these four individuals, the highest and lowest fractions of mapped *Y. pestis* reads were detected for Rise509 and Rise397, respectively. Interestingly, this relative pattern is also observed in the original study by Rasmussen et al. (2015). This observation serves as a general confirmation of the results presented in Rasmussen et al. (2015) and implies that the detection of this disease can be reproduced in most cases from different teeth of an infected skeleton. Both tooth dentine and cementum show evidence of *Y. pestis* infection except for Rise397 (a poorly preserved tooth sample) where the level in dentine is below our detection threshold (Figure 1). For the remaining three samples, dentine actually seems to “outperform” cementum 5–6 times, possibly because dentine (inner part of the root) is more directly in contact with the bloodstream in the pulp cavity. Interestingly, we could not detect *Y. pestis* DNA in the tooth from the Rise00 individual, contrasting the previously published results (Rasmussen et al., 2015). This result emphasizes that despite the general reproducibility, plague DNA levels can vary between teeth, implying that an apparent absence of plague DNA cannot be used to rule out an infection. More comparative analyses of ancient plague victims are needed to accurately assess the heterogeneity and detection sensitivity levels.

**Table 1:** Overview of the data and results

Sample	Trimmed reads	Plague reads	Normalized plague reads	Normalized Kraken reads	Damage C→T	Human DNA %	mtDNA haplogroup
Negative_cementum	12,326,411	77	62	1		22.3	
Negative_petrous	10,561,575	32	30	0		31.0	
Rise00_dentine	11,967,447	9	8	0		0.1	
Rise00_cementum	22,058,165	28	13	0		2.3	H5a1
Rise00_petrous	16,417,368	1	1	0		62.0	H5a1
Rise386_dentine	17,899,720	1,243	694	45	0.08	39.6	J1c1
Rise386_cementum	19,911,365	205	103	5	0.11	65.9	J1c1b1a
Rise386_petrous	23,943,845	3	1	0		68.8	J1c1b1a
Rise397_dentine	15,354,984	33	21	0		0.1	
Rise397_cementum	15,791,765	371	235	16	0.24	3.5	
Rise397_petrous	12,583,231	6	5	0		7.5	
Rise509_dentine	20,558,557	11,822	5,750	394	0.16	48.5	T2c1a2
Rise509_cementum	16,402,996	1,412	861	62	0.14	77.5	T2c1a2
Rise509_petrous	21,077,122	7	3	0		58.7	T2c1a2
Rise511_dentine	12,107,391	2,799	2,312	138	0.15	23.0	J2a2a
Rise511_cementum	19,841,683	1,384	698	56	0.14	31.8	J2a2a

*Trimmed reads*, total number of DNA reads per DNA library after adapter trimming, except for the negative control samples which are average values of 10 samples; *Plague reads* and *Normalized plague reads*, observed and normalized number of reads identified as *Y. pestis* based on reference genome mapping; *Normalized Kraken reads*, normalized number of reads identified as *Y. pestis* with the Kraken approach (Wood & Salzberg, 2014); *Damage C*→*T*, observed frequency of C→T transitions at the first position at the 5′ end of the mapped reads relative to the reference genome of *Y. pestis*; Human DNA %, the endogenous human DNA content; *mtDNA haplogroup*, the human mitochondrial haplogroup identified with haplogrep (Weissensteiner et al., 2016) in samples with sufficient data. Normalizations are based on mapped fractions assuming 10 million trimmed reads.

Importantly, none of the five petrous bones from the plague‐infected individuals had detectable levels of *Y. pestis* DNA (Table 1 and Figure 1). Despite the small sample size, this result strongly suggests that *Y. pestis* DNA does not enter the otic capsule. It is clear that the dense and structure of the otic capsule that facilitates good preservation of endogenous DNA seems to prevent the detection of blood‐borne pathogens perhaps due to having lower blood circulation levels compared to the other parts of the human skeleton. Alternatively, plague bacteria are present in high concentration in the saliva, explaining why it is detectable in the teeth.

For the seven samples with *Y. pestis* yields above baseline levels, we had sufficient data to test for DNA damage and we observe increased C→T deamination rates at the 5′ end (ranging from 8% to 24% at position 1) for all of them (Table 1 and Figure S1). This is a typical feature of ancient DNA molecules and confirms that the plague reads are authentic and of ancient origin. Although the absolute deamination rates were slightly lower than observed in the original paper, the relative C→T misincorporation rates (between‐sample differences) match the pattern observed by Rasmussen et al. (2015). Moreover, we observed a random distribution of DNA reads mapped to the *Y. pestis* genome (i.e., not clustered around conserved regions), further supporting the authenticity of the mapped DNA fragments. As a further confirmation of cross‐study comparability, we compared the human mtDNA haplogroups obtained from our shotgun data with the previously published data representing the same individuals (Allentoft et al., 2015). Ten samples (representing four individuals) had enough mtDNA coverage to reliably call the human mitochondrial haplogroups, and all showed the same haplogroup as in the original study (Table 1). DNA reads that mapped to the human reference genome also showed typical ancient DNA deamination profiles with slightly higher C→T transition rates observed in the petrous bones, as it was previously observed by Hansen et al. (2017).

### 3.2 The Kraken approach

As an alternative method of assessing the levels of *Y. pestis* DNA in the libraries, we used Kraken (Wood & Salzberg, 2014) for a taxonomic classification of the reads. We applied a confidence scoring threshold of 0.9, implying high classification precision but in the expense of sensitivity. This conservative confidence scoring threshold, removing ambiguous and unclassified k‐mers (e.g., due to DNA deamination damage), probably explains why the number of identified plague reads was smaller than in the mapping analysis (Table 1). However, the relative between‐sample distribution of identified *Y. pestis* sequences was highly similar with the two methods, confirming increased levels of *Y. pestis* DNA in teeth from Rise386, Rise397, Rise509, Rise511, and below the threshold for Rise00, and for all the petrous bones (Table 1).

We then investigated if the difference in *Y. pestis* DNA levels between teeth and petrous bones represented a general difference in microbial metagenomic composition in the two substrates. To test this in a homogenous sample set, representing the same age and preservation environment, we used data from the 10 plague‐negative control skeletons from Holmens Kirke. We combined the trimmed reads from all 10 tooth cementum samples into one group and all reads from the 10 petrous bones into another, yielding 123,264,109 and 105,615,747 reads for tooth and petrous bone, respectively. For comparative purposes, we then randomly down‐sampled the tooth dataset to 105,615,747 reads (matching the petrous bone dataset) and ran the Kraken classification on both datasets.

**Figure 2:**
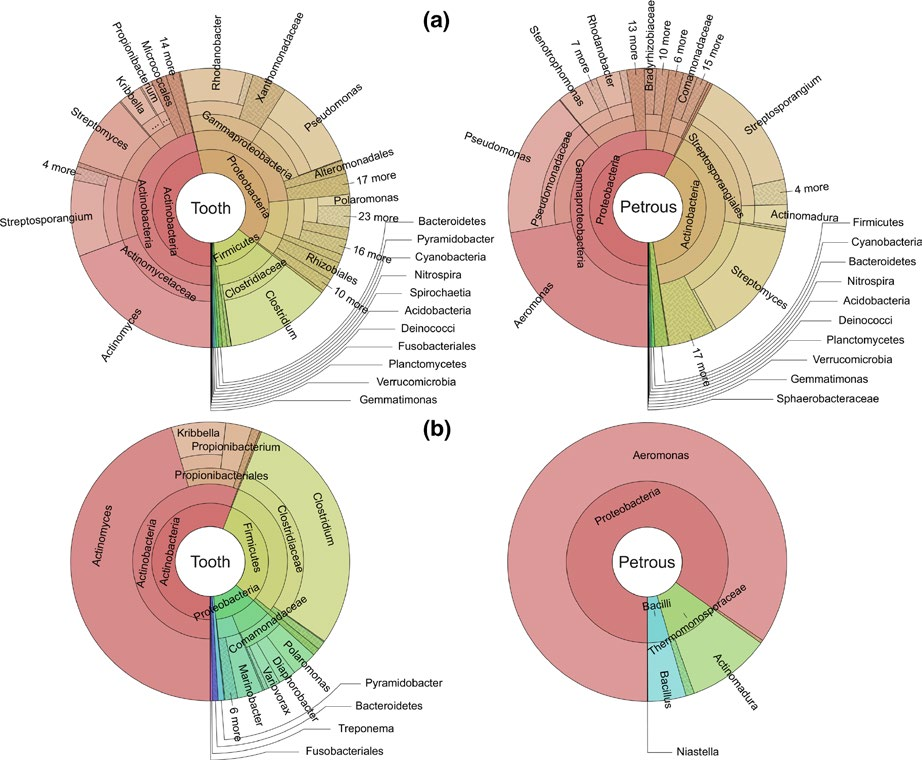
Abundance of bacterial genera in teeth and petrous bones. The total number of classified reads after removing rare (*n* < 10) classified reads was 394,874 and 315,166 in tooth and petrous datasets, respectively. (a) Classified bacterial genera in tooth (648 genera) and petrous bone (492 genera) datasets, which had at least 10 identified reads. (b) Classified bacterial genera displaying at least 10 times more reads in tooth compared to petrous bone (left) or in petrous bone compared to tooth (right)

These pooled metagenomic profiles are summarized in Table S2. As the vast majority of nonhuman reads were from bacteria, we focused on that domain in the downstream comparative analysis (Figure 2a). Moreover, we confined our comparative analysis to the genus level which was the lowest taxonomic level used by Wood and Salzberg (2014) for testing the sensitivity and accuracy of Kraken. We note that the accuracy of the Kraken classification depends on the diversity and number of reference sequences in the Kraken database. This, coupled with the short and heavily deaminated aDNA molecules, could easily result in false‐positive classification for some reads. We therefore removed classified genera identified with <10 reads, because such rare taxa may represent background “noise” rather than a real signal. Still, rigorous follow‐up analyses would be required to confirm each identified taxon as truly being represented by authentic aDNA in a sample. It is beyond the scope of the present study to provide an accurate and in‐depth taxonomic characterization of the microbial community in these tissues. We focus on the obvious comparative metagenomic differences and note that exact taxon matches (Table S2) should be interpreted with great caution.

In summary, 648 genera (with 394,874 sequences) were identified in the tooth dataset which is 30% more than the 492 genera (with 315,166 sequences) in the petrous bones. The general distribution pattern of major taxonomic bacterial groups is similar between both substrates (Figure 2a) with most classified reads from genera like *Pseudomonas*,* Streptomyces*,* Clostridium*, and *Aeromonas* which are all common in soil. Despite an overall qualitative similarity in the metagenomic profiles of tooth cementum and petrous bone, there are important differences. To emphasize this, we assessed the diversity of genera represented by a > 10‐fold difference in number of classified reads between the tooth and petrous bone datasets. These genera are essentially >10 times more likely to be identified in shotgun sequencing data from one substrate compared to the other. The result is summarized in Figure 2b. We identified 60 bacterial genera with >10 times more reads in the tooth cementum data than in the petrous bone data. In contrast, only eight bacterial genera were identified with >10 times more sequences in petrous bone compared to tooth cementum. (Table S3, Figure 2b). Among the 60 genera with a skewed presence in favor of tooth, many such as *Clostridium* and *Citrobacter* have both pathogenic species and ones commonly found in soil. Also, genera associated with human natural and pathogenic oral microbiome (http://www.homd.org/) such as *Actinomyces*,* Methanobrevibacter,* and *Streptococcus* were also much more abundant in the tooth‐derived dataset than in the petrous bone data. Among the eight genera being skewed in favor of petrous bone were *Bacillus* species, which are ubiquitous in nature, (Harwood, 2013) and *Aeromonas* species, which are found in freshwater and can be associated with a number of human diseases (Austin, 1996).

In summary, when considering bacterial genera that are clearly more common in one of the two substrates, tooth cementum samples display a much higher diversity than petrous bone. This observation matches the results from the *Y. pestis* experiment, confirming that the highly segregated distribution of bacterial DNA between tooth and petrous bone is not restricted to *Y. pestis* but represents a general metagenomic feature. A few of possible scenarios can account for this difference:

1. The otic capsule of the petrous bone is harder than the teeth roots, implying that only very little exogenous DNA will ever penetrate into this bone, both *premortem* and *postmortem*.
2. Teeth are more exposed than petrous bones to bacteria from the oral microbiome during an individual's lifetime. This explains the 100‐fold to 1,000‐fold higher number of classified reads of oral bacterial species such as *Actinomyces gerencseriae* (7,330‐fold), *Actinomyces sp oral taxon 448* (4,663‐fold), *Propionibacterium acidifaciens* (655‐fold), and *Tannerella forsythia* (471‐fold) in teeth samples compared to the petrous bones, where practically no reads from these species were detected (Table S4).
3. Differences in blood circulation and bone turnover rates in teeth and petrous bone (the otic capsule, more specifically) may account for the sharp contrast of *Y. pestis* DNA distribution between the two tissues, potentially representing a general difference in exposure to blood‐borne pathogens. For example, *Clostridium tetani*, a causative agent for tetanus, was identified with 2,130 sequences in the pooled tooth dataset and only 14 sequences in the pooled petrous bone data (Table S4). Most of these reads were derived from the same individual (sample M1) showing 614 times as many *C. tetani* classified reads in the tooth as in the petrous bone. While some of the tetanus sequences may originate from soil, the general absence of tetanus in the rest of the samples from the same cemetery indicates that the individual was indeed infected by this blood‐borne pathogen.
4. After inhumation, teeth are potentially more directly exposed to the soil (and thus soil bacterial DNA—both modern and ancient) compared to the otic capsule inside the petrous bones. This could explain the higher diversity of typical soil bacteria observed in the teeth.

### 3.3 Authenticity

To test for aDNA authenticity, we selected a few of the most abundant bacterial species with a skewed representation (Figure 2b). We mapped the trimmed DNA reads to the relevant reference genomes and this allowed us to assess the DNA damage patterns. The pathogen bacterium *Clostridium tetani* and the oral bacteria *Actinomyces gerencseriae* and *Actinomyces sp oral taxon 448* were, in the Kraken analysis, respectively, represented with 614‐fold, 7,330‐fold, and 4,663‐fold as many DNA sequences in the pooled tooth data compared to the pooled petrous bone data. When mapping the data to the reference genomes of these species, the mapped sequences showed typical aDNA deamination damage profiles with increased C→T transition rates at the first position of sequenced DNA fragments (Figure S2). However, in case of the petrous bone samples, either very few reads were mapped to the reference genomes (*C. tetani*—only 80 reads), preventing an assessment of damage, or the mapped sequences did not show deamination profiles (Figure S2 and Table S5). This suggests that most sequences mapping to above‐mentioned pathogenic and oral microbiome species are authentic and ancient when obtained from teeth, while the few reads of these species obtained from the petrous bones are likely to be false positives or modern contamination.

Likewise, we mapped and assessed the damage profiles of two bacteria classified with many more reads in the petrous bones than in teeth; *Aeromonas rivuli* and *Actinomadura madurae* reads were skewed 6.8‐fold and 2.9‐fold, respectively, in favor of the petrous bone data. DNA reads from petrous bones that mapped to these two species did not show convincing damage patterns (0.6% *A. rivuli* and 1.4% *A*. *madurae* C→T transition rates at position 1) despite a very high number of mapped reads (Figure S2). Interestingly, although the number of *A. rivuli* and *A. madurae* reads in the tooth dataset was much smaller, these displayed higher levels of DNA deamination damage (1.6% *A. rivuli* and 2.2% *A. madurae* C→T transition rates at position 1) (Figure S2 and Table S5).

Finally, in order to exclude any possible bias from potential background laboratory contamination, we also shotgun sequenced four extraction blanks. From these, only 32 bacterial species were classified, of which the vast majority were not among the ones with a skewed abundance, as discussed above (Table S6). Regardless, in the case of contamination, one would expect a random or perhaps an ubiquitous presence of such contaminant and it should not be able to drive the highly substrate‐specific pattern we have described.

Although these analyses do not represent an exhaustive quantitative comparison between all data and taxa identified in the two substrates, it is clear that the bacterial DNA obtained from tooth seems to be authentic and has a more ancient origin. In contrast, among the few bacterial species identified as more common in petrous bone, the absence (or very low level) of DNA damage indicates a higher degree of false‐positive matches because of poor database coverage, or a higher influence of modern bacterial DNA. The latter can be a direct effect of having very little ancient bacterial DNA in the extract, allowing low background levels of modern bacterial DNA to be built into the library. It is worth mentioning that there are of course differences in metagenomic composition between and within individuals. Apart from the obvious differences such as the burial location and soil microenvironment, another major factor could be the preservation state of the teeth and petrous bones. As one might expect, in relatively badly preserved petrous bone samples (with degraded and porous otic capsule), the diversity of bacterial taxa could be much higher.

## 4 CONCLUSION

Our study has contributed with several insights of relevance to the aDNA scientific community. First of all, finding *Yersinia pestis* DNA in an ancient skeleton is reproducible between different teeth (in four of five cases). This is an important observation for diagnostic purposes. Second, we find no traces of *Y. pestis* DNA in the petrous bone implying that the evolutionary and epidemic history of this bacterium cannot be studied genetically from this skeletal element. Consequently, if the skeletons in Rasmussen et al. (2015) had originally been sampled from the petrous bones, like in most ancient human genomic studies published in recent times, the discovery of these prehistoric plague infections would not have been made at that time. Our results show that this difference is not limited to *Y. pestis* but represents a general difference in the metagenomic profiles, where petrous bones (otic capsules) display little to none authentic ancient exogenous DNA. We emphasize that when identifying ancient pathogen DNA in a skeletal element, rigorous follow‐up analyses are needed to thoroughly address the authenticity and exact taxonomic origin of the reads (e.g., Rasmussen et al., 2015). A bioinformatical screening with, for example, Kraken is only the first exploratory step to identify interesting sequences that are candidates for further scrutiny. Nonetheless, our results point to the simple conclusion that if a skeleton has been sampled from the tooth, the chance of finding an interesting microbial signal is much higher than when using the petrous bone from the same individual. This is highly relevant when establishing the sampling strategy for any project that involves aDNA. Targeting the petrous bone is an efficient means to maximize the endogenous DNA content from an ancient individual, but it clearly comes with a cost. In that context, it is important to consider that tooth cementum, if well preserved and sampled correctly (Damgaard et al., 2015), can often yield an endogenous DNA content that is almost as high as in the petrous bone (Hansen et al., 2017), while also providing an authentic and diverse ancient metagenomic profile.

## CONFLICT OF INTEREST

None declared.

## AUTHOR CONTRIBUTIONS

M.E.A. initiated and led study; A.M. and M.E.A. designed study;A.M. and H.B.H. produced the data;A.M. and S.R. analyzed the data;A.M., S.R., M.S., E.W., and M.E.A. interpreted results;A.M. and M.E.A. wrote the manuscript with contributions from all authors; and V.M., A.K., A.E., L.Y., A.K., L.V., L.S., and N.L. excavated, curated, sampled and/or described, and analyzed skeletons. All authors contributed to interpretation of data.
