# Supplementary material for: Ancient pathogen DNA in human teeth and petrous bones
Source: Ecol Evol. 2018 Feb 26;8(6):3534–42. doi: 10.1002/ece3.3924 (PMC5869295; doi:10.1002/ece3.3924)

Figure S1. DNA deamination plots representing *Y. pestis DNA* obtained from dentine and cementum from teeth of four re-sampled skeletons previously tested positive for *Y. pestis* DNA in the cementum layer (Rasmussen et al. 2015). The x-axis shows the nucleotide position in the sequenced read. The C→T transition rates (as compared to the *Y. pestis* reference genome) are shown with red lines and the high levels observed here confirm that this is ancient DNA. Damage plots are not shown for Rise00 (Dentine + Cementum), Rise397 (Dentine) and all petrous bones because the *Y. pestis* levels in these were below our detection level (see Figure 1, main text).


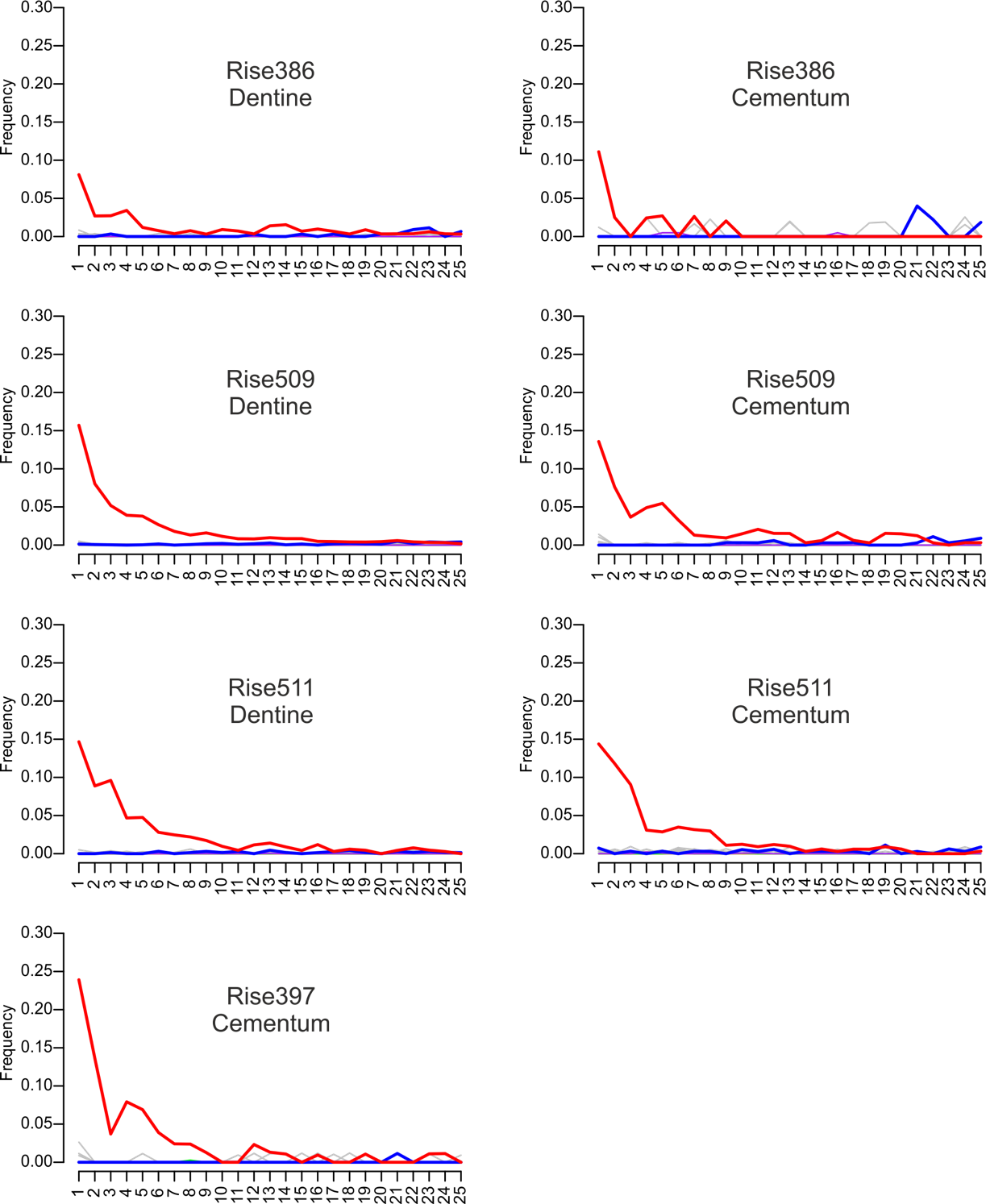


Figure S2. Damage plots of DNA reads from petrous bone and tooth datasets that were mapped to five bacterial species with highly different number of classified reads between the two datasets. The x-axis shows the nucleotide position in the sequenced read. The C→T transition rates are shown with red lines. Three bacterial species (*Clostridium tetani* and the oral bacteria *Actinomyces gerencseriae* and *Actinomyces sp oral taxon 448*) presented here have over 500-fold higher classified read in teeth samples compared with the petrous bones. DNA fragments from teeth samples clearly show typical ancient DNA C→T deamination pattern in contrast to the ones from the petrous bones. Interestingly, the two other species (*Aeromonas rivuli* and *Actinomadura madurae*) presented here that have respectively 6.8-fold and 2.9-fold more classified fragments in the petrous data do not show high levels of ancient DNA C→T deamination rates, indicating that most of the classified reads might be false positives and non-ancient.


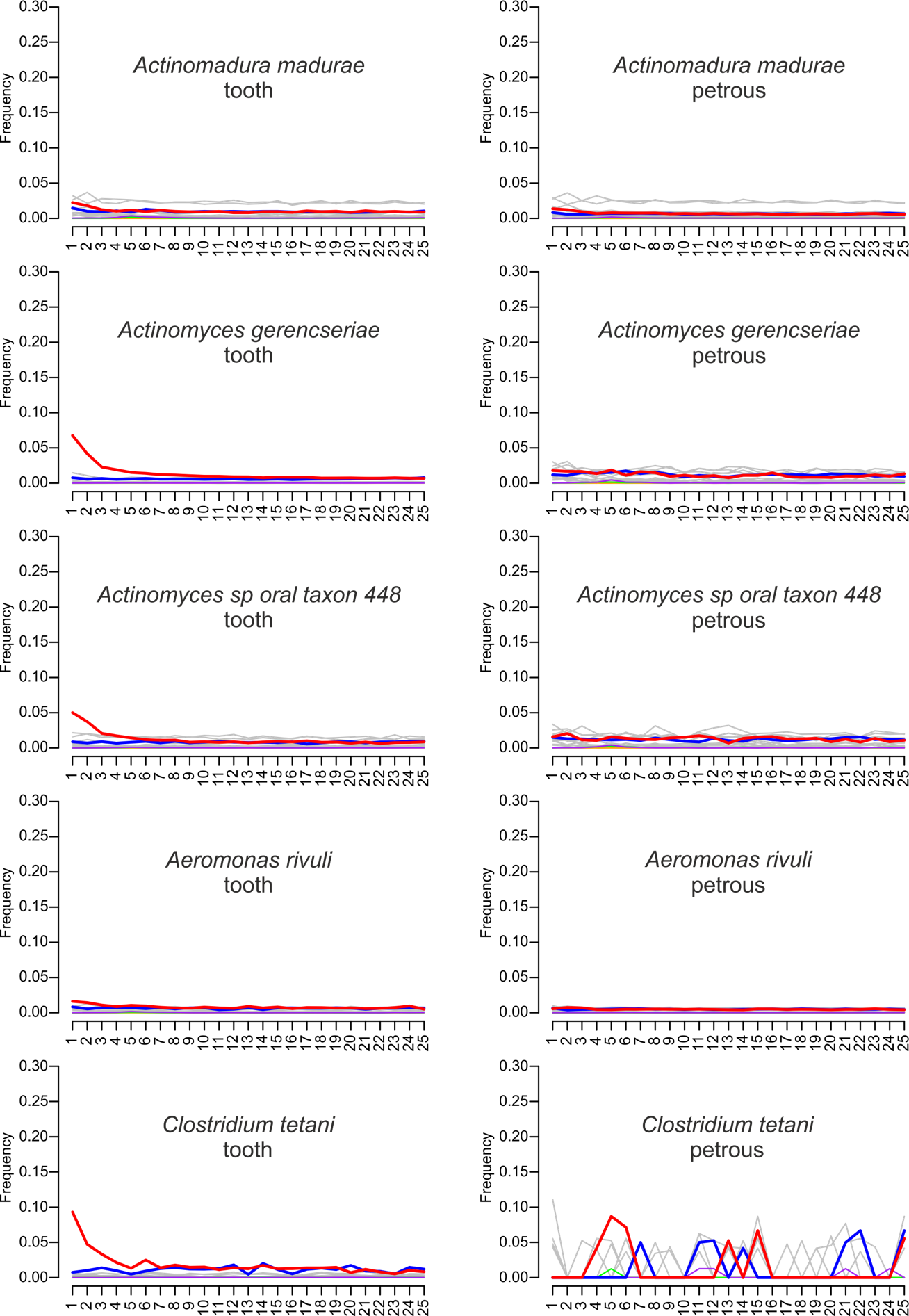

Supplement: Supplementary file 1 [file ECE3-8-3534-s001.docx]
